# Relationship Between Emotional Intelligence And Attitude Towards Peer’s Success And Failure In Adolescence And Youth

**DOI:** 10.1192/j.eurpsy.2022.2231

**Published:** 2022-09-01

**Authors:** N. Chernus, A. Serdakova, O. Karabanova, N. Korosteleva, N. Kurdyukova

**Affiliations:** The I.M. Sechenov First Moscow State Medical University: Moscow, Russia, The Outpatient Care Department, Moscow, Russian Federation

## Abstract

**Introduction:**

The problems of the attitude to success and failure are relevant at the present time.

**Objectives:**

study allows to describe and compare the indicators of emotional intelligence and the types of attitudes toward success and failure of a peer in adolescence and youth

**Methods:**

110 students and 90 teenagers were studied. T.V. Beskova’s methods “Attitude to the success and/or failure of a peer” in adolescence and adolescence (adapted technique by T.V. Beskova), N. Hall’s method of evaluating “emotional intelligence” (EQ questionnaire).

**Results:**

The “Relationship to Peer Success and Failure” methodology showed differences between adolescence and adolescence in scales: “peer joy” U = 0.016 (p≤0,05), “desire to achieve the same” U = 0.008 (p≤0,01), U = 0.027 “envy” (p≤0,05) and “passive” U = 0.006 (p≤0,01). The EQ questionnaire showed statistically significant differences in scales as “managing one’s emotions” U = 0.007 (p≤0,01), “self-motivation” U = 0.006 (p≤0,01). There are age differences in the types of attitudes to peer success and failure and the specifics of emotional intelligence in older adolescence and adolescence. Spearman rank correlation coefficient (r = 0.48) there is a relationship between the type of relationship to peer success and failure and emotional intelligence in adolescence and adolescence

**Conclusions:**

Thus, the study showed the features of personal factors in relation to the success and failure of a peer in adolescence and adolescence.

**Disclosure:**

No significant relationships.

